# Sample Ascertainment and Recruitment Sources in the Accelerating Medicines Partnership Schizophrenia Program

**DOI:** 10.1093/schizbullopen/sgaf013

**Published:** 2025-08-25

**Authors:** Jean Addington, Amy Shalev, Lu Liu, Cari Jahraus, Monica Chu, Emily Farina, Paolo Fusar Poli, Patricia J Marcy, Angela R Nunez, Monica E Calkins, Luis Alameda, Celso Arango, Owen Borders, Sylvain Bouix, Nicholas J K Breitborde, Matthew R Broome, Kristin S Cadenhead, Ricardo E Carrion, Rolando I Castillo-Passi, Eric Yu Hai Chen, Jimmy Choi, Michael J Coleman, Philippe Conus, Cheryl M Corcoran, Covadonga M Diaz-Caneja, Lauren M Ellman, Pablo A Gaspar, Carla Gerber, Louise Birkedal Glenthøj, Leslie E Horton, Christy Lai Ming Hui, Joseph Kambeitz, Lana Kambeitz-Ilankovic, Tina Kapur, Sinead Kelly, Melissa J Kerr, Matcheri S Keshavan, Minah Kim, Sung-Wan Kim, Nikolaos Koutsouleris, Jun Soo Kwon, Kerstin Langbein, Kathryn E Lewandowski, Daniel Mamah, Daniel H Mathalon, Vijay A Mittal, Catalina Mourgues, Merete Nordentoft, Ofer Pasternak, Godfrey D Pearlson, Nora Penzel, Jesus Perez, Diana O Perkins, Albert R Powers, Jack Rogers, Fred W Sabb, Jason Schiffman, Johanna Seitz-Holland, Jai L Shah, Steven M Silverstein, Stefan Smesny, William S Stone, Gregory P Strauss, Judy L Thompson, Rachel Upthegrove, Swapna Verma, Jijun Wang, Daniel H Wolf, Alison R Yung, Tianhong Zhang, Lauren Addamo, Kate Buccilli, Sophie Todd, Dominic Dwyer, Carrie E Bearden, John M Kane, Patrick D McGorry, Rene S Kahn, Martha E Shenton, Scott W Woods, Accelerating Medicines Partnership® Schizophrenia Nelson Barnaby

**Affiliations:** Department of Psychiatry, Hotchkiss Brain Institute, University of Calgary, Calgary, AB, T2N 4Z6, Canada; Department of Psychiatry, Hotchkiss Brain Institute, University of Calgary, Calgary, AB, T2N 4Z6, Canada; Department of Psychiatry, Hotchkiss Brain Institute, University of Calgary, Calgary, AB, T2N 4Z6, Canada; Department of Psychiatry, Hotchkiss Brain Institute, University of Calgary, Calgary, AB, T2N 4Z6, Canada; Department of Psychiatry, Hotchkiss Brain Institute, University of Calgary, Calgary, AB, T2N 4Z6, Canada; Department of Psychiatry, Yale University School of Medicine, New Haven, CT 06519, United States; Department of Psychosis Studies, Institute of Psychiatry, Psychology and Neuroscience, King’s College, London, SE58AF, United Kingdom; Department of Brain and Behavioral Sciences, University of Pavia, 27100 Pavia, Italy; Northwell, Glen Oaks, NY 11004, United States; Department of Psychiatry, Yale University School of Medicine, New Haven, CT 06519, United States; Connecticut Mental Health Center, New Haven, CT 06519, United States; Department of Psychiatry, Perelman School of Medicine, University of Pennsylvania, Philadelphia, PA 19063, United States; Department of Psychosis Studies, Institute of Psychiatry, Psychology and Neuroscience, King’s College, London, SE58AF, United Kingdom; National Psychosis Unit, South London and Maudsley NHS Foundation Trust, London, SE58AZ, United Kingdom; Service of General Psychiatry, Treatment and Early Intervention in Psychosis Program, Lausanne University Hospital (CHUV), 1003 Lausanne, Switzerland; Department of Child and Adolescent Psychiatry, Institute of Psychiatry and Mental Health, Hospital General Universitario Gregorio Marañón, IiSGM, CIBERSAM, Instituto de Salud Carlos III, School of Medicine, Universidad Complutense, 28009 Madrid, Spain; Department of Psychiatry, Brigham and Women’s Hospital, Harvard Medical School, Boston, MA 02115, United States; Department of Software Engineering and Information Technology, École de Technologie Supérieure, Montréal, QC, H3C 1K3, Canada; Department of Psychiatry and Behavioral Health, Early Psychosis Intervention Center (EPICENTER), College of Medicine, The Ohio State University Wexner Medical Center, Columbus, OH 43210, United States; Institute for Mental Health, University of Birmingham, Birmingham, B152TT, United Kingdom; Early Intervention for Psychosis Services, Birmingham Women’s and Children’s NHS Foundation Trust, Birmingham, B138QE, United Kingdom; University of California, San Diego, La Jolla, CA 92104, United States; Northwell, Glen Oaks, NY 11004, United States; Donald and Barbara Zucker School of Medicine at Hofstra/Northwell, Hempstead, NY 11549, United States; Institute of Behavioral Science, Feinstein Institutes of Medical Research, Manhasset, NY 11030, United States; Department of Psychiatry, University of Chile, 8330111 Santiago, Chile; Department of Neurology and Psychiatry, Clínica Alemana - Universidad del Desarrollo, 7610658 Santiago, Chile; Department of Psychiatry, School of Clinical Medicine, LKF Faculty of Medicine, University of Hong Kong, Hong Kong SAR, China; Olin Neuropsychiatry Research Center, Hartford HealthCare Behavioral Health Network, Hartford, CT 06106, United States; Department of Psychiatry, Brigham and Women’s Hospital, Harvard Medical School, Boston, MA 02115, United States; General Psychiatry Service, Treatment and Early Intervention in Psychosis Program (TIPP–Lausanne), Lausanne University Hospital and University of Lausanne, 1003 Lausanne, Switzerland; Department of Psychiatry, Icahn School of Medicine at Mount Sinai, New York, NY 10029, United States; Department of Child and Adolescent Psychiatry, Institute of Psychiatry and Mental Health, Hospital General Universitario Gregorio Marañón, IiSGM, CIBERSAM, Instituto de Salud Carlos III, School of Medicine, Universidad Complutense, 28009 Madrid, Spain; Department of Psychology & Neuroscience, Temple University, Philadelphia, PA 19122-6085, United States; Department of Psychiatry, University of Chile, 8330111 Santiago, Chile; Prevention Science Institute, University of Oregon, Eugene, OR 97401, United States; Oregon Research Institute, Springfield, OR 97477, United States; VIRTU Research Group, Mental Health Copenhagen, University of Copenhagen, 2900 Copenhagen, Denmark; Department of Psychology, University of Copenhagen, 1353 Copenhagen, Denmark; Department of Psychiatry, University of Pittsburgh School of Medicine, Pittsburgh, PA 15213, United States; Department of Psychiatry, School of Clinical Medicine, LKF Faculty of Medicine, University of Hong Kong, Hong Kong SAR, China; Department of Psychiatry, Faculty of Medicine and University Hospital Cologne, University of Cologne, 50937 Cologne, Germany; Department of Psychiatry, Faculty of Medicine and University Hospital Cologne, University of Cologne, 50937 Cologne, Germany; Faculty of Psychology and Educational Sciences, Department of Psychology, Ludwig-Maximilian-University, 80802 Munich, Germany; Department of Radiology, Brigham and Women’s Hospital and Harvard Medical School, Boston, MA 02115, United States; Department of Psychiatry, Brigham and Women’s Hospital, Harvard Medical School, Boston, MA 02115, United States; Orygen, Parkville, Victoria, 3052, Australia; Department of Psychiatry, Beth Israel Deaconess Medical Center, Harvard Medical School, Boston, MA 02115, United States; Department of Neuropsychiatry, Seoul National University Hospital, Seoul 03082, South Korea; Department of Psychiatry, Seoul National University College of Medicine, Seoul 03080, South Korea; Department of Psychiatry, Chonnam National University Medical School, Gwangju 61469, South Korea; Mindlink, Gwangju Bukgu Mental Health Center, Gwangju 61005, South Korea; Department of Psychosis Studies, Institute of Psychiatry, Psychology and Neuroscience, King’s College, London, SE58AF, United Kingdom; Department of Psychiatry and Psychotherapy, Ludwig-Maximilian-University, 80336 Munich, Germany; Max Planck Institute of Psychiatry, 80804 Munich, Germany; Department of Psychiatry, Seoul National University College of Medicine, Seoul 03080, South Korea; Department of Psychiatry, Hanyang University Hospital, Seoul 04763, South Korea; Department of Psychiatry and Psychotherapy, Jena University Hospital, 07743 Jena, Germany; Psychotic Disorders Division, McLean Hospital, Belmont, MA 02478, United States; Department of Psychiatry, Harvard Medical School, Boston, MA 02115, United States; Department of Psychiatry, Washington University Medical School, St. Louis, MO 63110, United States; Department of Psychiatry and Behavioral Sciences and Weill Institute for Neurosciences, University of California, San Francisco, San Francisco, CA 94121, United States; Mental Health Service 116D, Veterans Affairs San Francisco Health Care System, San Francisco, CA 94121, United States; Department of Psychology, Northwestern University, Evanston, IL 60201, United States; Department of Psychiatry, Yale University School of Medicine, New Haven, CT 06519, United States; VIRTU Research Group, Mental Health Copenhagen, University of Copenhagen, 2900 Copenhagen, Denmark; Department of Clinical Medicine, University of Copenhagen, 2200 Copenhagen, Denmark; Department of Psychiatry, Brigham and Women’s Hospital, Harvard Medical School, Boston, MA 02115, United States; Department of Radiology, Brigham and Women’s Hospital and Harvard Medical School, Boston, MA 02115, United States; Department of Psychiatry, Massachusetts General Hospital and Harvard Medical School, Boston, MA 02115, United States; Department of Psychiatry, Yale University School of Medicine, New Haven, CT 06519, United States; Olin Neuropsychiatry Research Center, Hartford Hospital, Hartford, CT 06106, United States; Department of Psychiatry, Brigham and Women’s Hospital, Harvard Medical School, Boston, MA 02115, United States; Department of Psychiatry and Psychotherapy, Ludwig-Maximilian-University, 80336 Munich, Germany; Department of Psychiatry, Massachusetts General Hospital and Harvard Medical School, Boston, MA 02115, United States; CAMEO, Early Intervention in Psychosis Service, Cambridgeshire and Peterborough NHS Foundation Trust, Cambridge, Cambridgeshire, CB4 1PX, United Kingdom; Department of Medicine, Institute of Biomedical Research (IBSAL), Universidad de Salamanca, 37007 Salamanca, Spain; Department of Psychiatry, University of North Carolina at Chapel Hill, Chapel Hill, NC 27514, United States; Department of Psychiatry, Yale University School of Medicine, New Haven, CT 06519, United States; Connecticut Mental Health Center, New Haven, CT 06519, United States; Institute for Mental Health, University of Birmingham, Birmingham, B152TT, United Kingdom; Centre for Human Brain Health, University of Birmingham, Birmingham, B152TT United Kingdom; Prevention Science Institute, University of Oregon, Eugene, OR 97401, United States; Department of Psychological Science, University of California, Irvine, Irvine, CA 92697-7085, United States; Department of Psychiatry, Brigham and Women’s Hospital, Harvard Medical School, Boston, MA 02115, United States; Department of Psychiatry, Massachusetts General Hospital and Harvard Medical School, Boston, MA 02115, United States; Douglas Research Centre, Montreal, QC, H4H 1R3 Canada; Department of Psychiatry, McGill University, Montreal, QC H3A 1A1, Canada; Department of Psychiatry, University of Rochester Medical Center, Rochester, NY 14642, United States; Department of Psychiatry and Psychotherapy, Jena University Hospital, 07743 Jena, Germany; Department of Psychiatry, Beth Israel Deaconess Medical Center, Massachusetts Mental Health Center and Harvard Medical School, Boston, MA 02115, United States; Department of Psychology, University of Georgia, Athens, GA 30602, United States; Departments of Psychiatry and Neuroscience, University of Rochester Medical Center, Rochester, NY 14642, United States; Institute for Mental Health, University of Birmingham, Birmingham, B152TT, United Kingdom; Department of Psychiatry, University of Oxford, Oxford, OX37JX, United Kingdom; Institute of Mental Health, Singapore 539747, Singapore; Duke-NUS Medical School, Singapore 169857, Singapore; Shanghai Mental Health Center, Shanghai Jiaotong University School of Medicine, Shanghai 200030, China; Department of Psychiatry, Perelman School of Medicine, University of Pennsylvania, Philadelphia, PA 19063, United States; Institute of Mental and Physical Health and Clinical Translation (IMPACT), Deakin University, Geelong, Victoria, 3220, Australia; School of Health Sciences,University of Manchester, Manchester, M139PL, United Kingdom; Department of Psychiatry, Shanghai Mental Health Center, Shanghai Jiaotong University School of Medicine, Shanghai 200030, China; Orygen, Parkville, Victoria, 3052, Australia; Centre for Youth Mental Health, The University of Melbourne, Parkville, Victoria, 3052 Australia; Orygen, Parkville, Victoria, 3052, Australia; Centre for Youth Mental Health, The University of Melbourne, Parkville, Victoria, 3052 Australia; Orygen, Parkville, Victoria, 3052, Australia; Centre for Youth Mental Health, The University of Melbourne, Parkville, Victoria, 3052 Australia; Orygen, Parkville, Victoria, 3052, Australia; Centre for Youth Mental Health, The University of Melbourne, Parkville, Victoria, 3052 Australia; Departments of Psychiatry and Biobehavioral Sciences and Psychology, Semel Institute for Neuroscience and Human Behavior; University of California, Los Angeles, Los Angeles, CA 90095, United States; Department of Psychiatry, Donald and Barbara Zucker School of Medicine, Hempstead, NY 11549, United States; Feinstein Institute for Medical Research, Manhasset, NY 11030, United States; Orygen, Parkville, Victoria, 3052, Australia; Centre for Youth Mental Health, The University of Melbourne, Parkville, Victoria, 3052 Australia; Department of Psychiatry, Icahn School of Medicine at Mount Sinai, New York, NY 10029, United States; Department of Psychiatry, Massachusetts General Hospital and Harvard Medical School, Boston, MA 02115, United States; Department of Psychiatry and Radiology, Brigham and Women’s Hospital and Harvard Medical School, Boston, MA 02115, United States; Department of Psychiatry, Yale University School of Medicine, New Haven, CT 06519, United States; Orygen, Parkville, Victoria, 3052, Australia; Centre for Youth Mental Health, The University of Melbourne, Parkville, Victoria, 3052 Australia

**Keywords:** clinical high risk, community controls, psychosis, ascertainment, recruitment, AMP SCZ

## Abstract

**Background:**

This paper presents the recruitment sources of clinical high-risk (CHR) and community controls (CC) from the Accelerating Medicines Partnership Schizophrenia (AMP SCZ) program, which aims to study various clinical variables and biomarkers in 2040 CHR and 652 CC participants.

**Methods:**

A total of 1640 CHR and 514 CC had recruitment source data. The Positive Symptoms and Diagnostic Criteria for the Comprehensive Assessment of At-Risk Mental States Harmonized with the SIPS was utilized to assess CHR criteria and severity of attenuated psychotic symptoms (APSs), and the Global Functioning: Social Scale was used for social functioning. Participants were recruited through various methods, including referrals from healthcare providers, schools, and community agencies, and self-referrals via outreach efforts and advertising.

**Results:**

Participants were recruited from 13 different sources, with self-referral being the most common for both CHR and CC. Other notable sources included child and youth services and psychiatric hospitals and departments. Regional differences in recruitment patterns were observed across continents. Differences in age, APS, and social functioning for CHR participants were examined in the top 5 recruitment sources. Overall, self-referred individuals were typically older, with less severe APS and higher levels of functioning, whereas those from adult community mental health services had poorer functioning and more severe APS. The remaining recruitment groups fell between these 2 extremes.

**Conclusion:**

This paper highlights the diverse recruitment sources for the AMP SCZ program. Self-referral was a significant source, particularly in North America, reflecting changing help-seeking behaviors influenced by the internet and social media. The findings underscore the importance of understanding recruitment sources to optimize future CHR research.

## Introduction

The Accelerating Medicines Partnership Schizophrenia (AMP SCZ) program consists of 2 large clinical networks: the Psychosis-Risk Outcomes Network (ProNET) and the Prediction Scientific Global Consortium (PRESCIENT); plus a data center, the Psychosis Risk Evaluation, Data Integration and Computational Technologies: Data Processing, Analysis, and Coordination Center. Together, the 2 networks have collected data from 2040 participants who are at clinical high-risk for developing psychosis (CHR) and 652 community controls (CC) enrolled in 43 sites across North America, Australia, Europe, South America, and Asia. The AMP SCZ program has 2 main aims. First, to create algorithms that will predict the clinical trajectories and outcomes of CHR individuals, and second, to develop and test tools for testing new pharmacological treatments for this CHR population.^1^^,^^2^ Comprehensive details about the complete AMP SCZ program can be found at www.ampscz.org. Additionally, a series of open access papers describing the modalities under study are published in a special issue of “Schizophrenia”.

One of the challenges faced by CHR researchers has been the accurate ascertainment of CHR participants. Higher rates of transition to psychosis are desirable in studies, as this increases the statistical power necessary to draw conclusions about the development of psychotic disorders from this research. Young people at CHR for psychosis have heterogeneous outcomes, with a minority of these individuals making the transition to a diagnosed psychotic disorder.^3^ Research addressing recruitment methods in this population suggests that higher transition rates are achieved when participants are recruited from help-seeking and clinical populations that are risk-enriched because of the accumulation of risk factors for psychosis.^4^

Accordingly, recruiting from the general population results in low-risk enrichment and overall reduction of risk for psychosis.^4^ Furthermore, other biases may be attributable to ascertainment, with 1 study finding that, compared to a screening method in a mental health clinic, a recruitment strategy for a second opinion from psychiatrists, psychologists, family doctors, outreach services, counseling services, or self-referral resulted in lower transition rates and was biased to recruitment of younger males.^5^ Currently, recruitment strategies in the psychosis risk field remain unstandardized, with recruitment sources highly varied.^6–10^

The aim of this paper is to present details of how participants, both CHR and CC, in the AMP SCZ program were ascertained. Second, differences between recruitment sources for CHR and CC participants and among different continents will be examined. Third, since attenuated psychotic symptoms (APSs) and social functioning have in earlier studies been predictive of later transition to psychosis,^11–13^ we will examine whether the severity of APSs and social functioning deficits vary among the different recruitment sources and whether there are demographic differences such as age or sex across recruitment sources.

Over the course of the AMP SCZ program, data will be made available to the larger research community through the National Institute of Mental Health (NIMH) Data Archive (NDA). Data for this paper were from the third AMP SCZ data release by the NDA and represents approximately 80% of the final baseline dataset.

## Methods

Further details of the methodology of the ascertainment and clinical domains of the AMP SCZ program are available in a recent paper^14^ and on the website www.ampscz.org. The standard operating procedures, “Clinical Data Acquisition”, are available in the section “For Scientists” using the link www.ampscz.org/scientists/*.*

The dataset used in this study was generated by the AMP SCZ Program^2^ and downloaded from the NIMH Data Archive (data release 3.0 doi:10.15154/81xe-k706).

### Participants

The samples for this paper are the 1640 CHR participants and 514 CC who had data available from the NIMH Data Archive release 3.0 on recruitment sources. Inclusion criteria for CHR and CC were ages 12-30 years and ability to provide written informed consent (parental/guardian consent obtained for participants ages <18 years). Clinical high-risk only inclusion criteria included: Comprehensive Assessment of At-Risk Mental States (CAARMS) defined (trait vulnerability; APSs; brief limited intermittent psychotic symptoms) or Structured Interview for Psychosis-Risk Syndromes (SIPS) defined (brief intermittent psychotic syndrome current progression; attenuated positive symptom syndrome current progression; genetic risk and deterioration current progression) diagnostic criteria for CHR, determined using the *P*ositive *SY*mptoms and Diagnostic Criteria for the *C*AARMS *H*armonized with the *S*IPS (PSYCHS) (see below). Antipsychotic medication exposure equivalent to a total lifetime haloperidol dose of >50 mg, estimated based on available information, or current antipsychotic medication at the time of baseline assessment was an exclusion criterion for all participants. Complete inclusion and exclusion criteria are presented in Table S1. The study was approved by institutional review boards at all ProNET and PRESCIENT sites. Table S2 presents the names and locations of the 43 sites.

### Measures

The PSYCHS is a semi-structured interview used to determine CHR criteria and the severity of APS for both the CAARMS^15^ and the SIPS.^16^ PSYCHS development has been described elsewhere.^17^^,^^18^ The PSYCHS consists of 15 APS, which can be used to generate APS severity ratings and diagnoses on both the SIPS and the CAARMS. See also the special issue in “Early Intervention in Psychiatry” for more details on the PSYCHS.^19^

Social functioning was assessed with the Global Functioning (GF): Social Scale developed by Cornblatt et al. to measure changes in social functioning across time in CHR participants.^20^^,^^21^ The GF: Social Scale rates peer relationships, conflict, and family involvement, and scores range from 1 (extreme dysfunction) to 10 (superior functioning).

The Clinical Ascertainment and Outcome Measures Team of the AMP SCZ program^2^^,^^14^ compiled a list of potential recruitment sources. These sources were reviewed by the lead investigators from the international sites to ensure that as many recruitment sources as possible were listed. Fifteen key sources of recruitment were noted, with each having several subcategories. These are listed in Table 1.

**Table 1:** Frequency of Recruitment Sources for Participants

**Recruitment source**	**CHR (*n* = 1640)**		**CC (*n* = 514)**	
	**Frequency**	**(%)**	**Frequency**	**(%)**
**Self-referred**	**599**	**(36.5)**	**344**	**(66.9)**
Self	548	(91.5)	234	(67.8)
Relative/caregiver	17	(2.8)	19	(5.5)
Friend/colleague/other participant/social or team leader	26	(4.3)	86	(24.9)
Other	8	(1.3)	6	(1.7)
**Child and youth services**	**391**	**(23.8)**	**2**	**(0.4)**
Mental health services	387	(99.0)	1	(50.0)
Non-mental health services	2	(0.5)	0	(0.0)
Youth Shelter, ie, homeless	0	(0.0)	1	(50.0)
Other	2	(0.5)	0	(0.0)
**Psychiatric hospital or department**	**208**	**(12.7)**	**1**	**(0.2)**
Psychiatrist	97	(46.6)	0	(0.0)
Inpatient	27	(13.0)	0	(0.0)
Other referral sources within the hospital	68	(32.7)	1	(100.0)
Emergency/urgent care	5	(2.4)	0	(0.0)
Other	11	(5.3)	0	(0.0)
**Another study**	**172**	**(10.5)**	**70**	**(13.6)**
**Adult community mental health services**	**79**	**(4.8)**	**1**	**(0.2)**
First episode program	25	(31.6)	0	(0.0)
Specialized call line for FEP or CHR	16	(20.3)	0	(0.0)
Other	38	(48.1)	1	(100.0)
**General population screening**	**50**	**(3.0)**	**19**	**(3.7)**
**Community agencies**	**49**	**(3.0)**	**1**	**(0.2)**
Mental health agency	43	(87.8)	0	(0.0)
Health center	4	(8.2)	0	(0.0)
Other	2	(4.1)	1	(100.0)
**Physician**	**32**	**(2.0)**	**0**	**(0.0)**
General practitioner	1	(3.1)	0	(0.0)
Psychiatrist	27	(84.4)	0	(0.0)
Pediatrician	4	(12.5)	0	(0.0)
**Non-physician**	**25**	**(1.5)**	**1**	**(0.2)**
Psychologist	18	(72.0)	1	(100.0)
Social worker	4	(16.0)	0	(0.0)
Other practitioner	3	(12.0)	0	(0.0)
**Post-secondary education**	**11**	**(0.7)**	**37**	**(7.2)**
Mental health services	5	(45.5)	0	(0.0)
General counseling	2	(18.2)	0	(0.0)
Health services	1	(9.1)	7	(18.9)
Other	3	(27.3)	30	(81.1)
**Grade school**	**7**	**(0.4)**	**2**	**(0.4)**
Guidance counseling	2	(28.6)	0	(0.0)
Psychology department	1	(14.3)	0	(0.0)
Teacher or other personnel	3	(42.9)	1	(50.0)
Other	1	(14.3)	1	(50.0)
**Consumer organization**	**3**	**(0.2)**	**0**	**(0.0)**
Youth mental health focused organizations	1	(33.3)	0	(0.0)
Other	2	(66.7)	0	(0.0)
**Other**	**14**	**(0.9)**	**36**	**(7.0)**
Registry of potential participants	13	(92.9)	14	(38.9)
Invited by program or research staff	1	(7.1)	22	(61.1)

Abbreviations: CHR, clinical high-risk; CC, community control.

### Procedures

Several different methods were used to recruit participants to the project. Many participants were help-seeking individuals referred by doctors and clinicians from a range of hospital- and community-based mental health practices and programs and from private practices. Likewise, referrals were sought from schools and post-secondary institutions as well as community agencies. Additionally, participants were family- and self-referred in response to community outreach, including targeted advertising on social and mainstream media. Other sources included other studies, general population screening, and consumer organizations. Community controls were explicitly recruited and referred to as control participants.

At screening, if the participant had been specifically referred to the study, the recruitment source was documented and clarified with the participant. Otherwise, the participant and/or family were asked how they heard of the research clinic or project.

For the PSYCHS, all raters underwent intensive training and met predetermined reliability standards to be certified. Intraclass correlations, based on initial data, demonstrate excellent inter-rater reliability between trainees and the gold standard for this measure.^14^ After screening, clinical vignettes were written about each participant and presented at conference calls to reach a consensus decision on symptom ratings and diagnosis. Vignettes included a detailed description of each of the fifteen PSYCHS APS items with discussion of the 4 PSYCHS measurement concepts (description, tenacity/source, distress, and interference) and symptom frequency.^17^

### Statistical analysis

Sociodemographic differences between CHR and CC groups were assessed at baseline using independent samples t-tests for continuous variables and chi-square tests for categorical variables. Frequencies and percentages were calculated to describe the distribution of CHR and CC participants by site, continent, and network (ProNET or PRESCIENT). Additional frequency analyses were conducted to compare the percentage of participants from each recruitment source between CHR and CC groups, as well as between CHR participants in the ProNET and PRESCIENT networks. For each recruitment source, the distribution of CHR participants was further analyzed by continent and by sex (female or male).

The means for age, total PSYCHS score, and GF: Social were calculated for each recruitment source among CHR participants. A chi-square test of independence was conducted to examine the association between sex (male vs female) and recruitment source.

To further explore differences across recruitment sources, a General Linear Model (GLM) was employed to assess variation in age, PSYCHS total scores, and GF: Social scores. While an Analysis of Variance (ANOVA) is a special type of GLM, the GLM framework offers greater flexibility by accommodating unbalanced data, non-normal distributions, and more complex model structures. The Tukey–Kramer test was used for post hoc comparisons. Since several of the recruitment sources had a very small proportion of CHR participants, for sufficient statistical power and comparability, only the top 5 recruitment sources were included in the GLM analysis. These 5 each represented at least 4.8% of the CHR sample and collectively represented 88.3% of the total CHR sample. A supplementary analysis, comparing the top 5 recruitment sources (based on frequency) to all other sources combined into a single category, was also conducted to evaluate whether the patterns observed in the top-source analysis were consistent across the broader sample.

## Results

### Demographics

There were more females than males in both the CHR and CC groups, with no significant difference in sex distribution between the groups: 1056 females (64.39%) and 584 males (35.61%) in the CHR group, and 319 females (61.46%) and 200 males (38.54%) in the CC group. CHR participants were slightly younger than CC participants, with a mean age of 21.2 years (SD = 4.0) compared to 21.6 years (SD = 3.6) in the CC group, a difference that was statistically significant (*P* < .05; Cohen’s *d* = 0.105). The majority of participants in both groups identified as Caucasian: 58.25% of CHR participants and 52.24% of CC participants. In the CHR group, 19.45% identified as Asian and 8.63% as Black, while in the CC group, 32.55% identified as Asian and 6.24% as Black. Further demographic details can be found in Table S3.

### Recruitment sources

**Figure 1:**
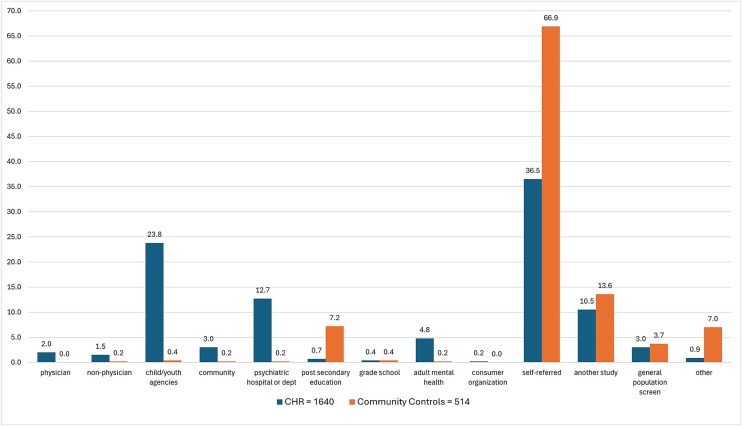
Percentages of CHR and CC Participants in Each of the Recruitment Sources.

**Figure 2:**
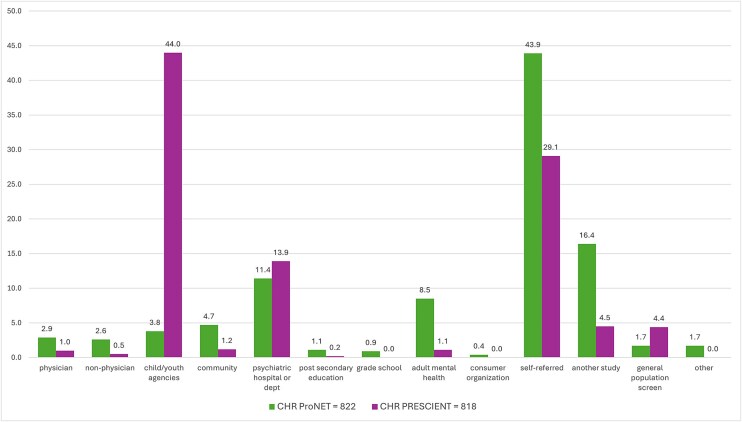
Percentages of CHR Participants from ProNET and PRESCIENT in Each of the Recruitment Sources.

Of the fifteen original recruitment sources, no participants were recruited through faith-based organizations or the judicial/police or criminal justice system. Clinical high-risk and CC participant numbers from the remaining 13 categories, along with their respective subcategories, are presented in Table 1. The percentage distribution of CHR and CC participants across these recruitment sources is illustrated in Figure 1. For both CHR and CC groups, the most common recruitment source was self-referral. Detailed information on how self-referred individuals learned about the project is provided in Table 2. For the CHR group, the next most common sources were child and youth services, followed by psychiatric hospitals or psychiatric departments, and then by other studies. For the CC group, the primary additional recruitment sources were other studies and post-secondary institutions. A comparison of recruitment sources between the ProNET and PRESCIENT networks (Figure 2) showed that the majority of participants referred by child and youth services were enrolled through the PRESCIENT network.

**Table 2:** Breakdown of Recruitment Sources for Self-Referred

**Source**	**CHR (*n* = 599)**		**CC (*n* = 344)**	
	**Frequency**	**(%)**	**Frequency**	**(%)**
Program or another website	31	(5.2)	32	(9.3)
Community flyer or advert	99	(16.5)	88	(25.6)
Community online advert	443	(74.0)	154	(44.8)
Outreach event	3	(0.5)	21	(6.1)
Newspaper or journal	0	(0.0)	1	(0.3)
Unknown	23	(3.8)	48	(13.9)

Abbreviations: CHR, clinical high-risk; CC, community control.

Table 3 presents the breakdown of recruitment sources by continent for CHR participants. In the North American sites, the most common recruitment source was self-referral, followed by recruitment from other studies, with participants also referred from various other sources, albeit in much smaller numbers. In the Australian sites, child and youth services were the most common recruitment source, followed by self-referral. In the European sites, most participants were referred from psychiatric hospitals or psychiatric departments, followed by adult community mental health services and child and youth services. In the Asian sites, referrals were spread across 3 main sources: general population screening, psychiatric hospitals or psychiatric departments, and self-referral. Chile was the only site in South America, with a small sample of 10, most of whom were referred by a physician.

**Table 3:** Frequency of Recruitment Sources within Each Continent for Clinical High-Risk Participants

**Referral source**	**North America (*n* = 669)**		**Australia (*n* = 576)**		**Europe (*n* = 275)**		**Asia (*n* = 110)**		**South America (*n* = 10)**	
	**Frequency (%)**		**Frequency (%)**		**Frequency (%)**		**Frequency (%)**		**Frequency (%)**	
Self-referred	354	(52.9)	201	(34.9)	11	(4.0)	30	(27.3)	3	(30.0)
Child and youth services	16	(2.4)	336	(58.3)	38	(13.8)	1	(0.9)	0	(0.0)
Psychiatric hospital or department	46	(6.9)	0	(0.0)	128	(46.5)	33	(30.0)	1	(10.0)
Another study	129	(19.3)	35	(6.1)	7	(2.5)	1	(0.9)	0	(0.0)
Adult community mental health services	12	(1.8)	0	(0.0)	63	(22.9)	4	(3.6)	0	(0.0)
General population screening	7	(1.0)	1	(0.2)	7	(2.5)	35	(31.8)	0	(0.0)
Community agencies	36	(5.4)	2	(0.3)	6	(2.2)	5	(4.5)	0	(0.0)
Physician	18	(2.7)	0	(0.0)	7	(2.5)	1	(0.9)	6	(60.0)
Non-physician	19	(2.8)	0	(0.0)	6	(2.2)	0	(0.0)	0	(0.0)
Post-secondary education	8	(1.2)	1	(0.2)	2	(0.7)	0	(0.0)	0	(0.0)
Grade school	7	(1.0)	0	(0.0)	0	(0.0)	0	(0.0)	0	(0.0)
Consumer organization	3	(0.4)	0	(0.0)	0	(0.0)	0	(0.0)	0	(0.0)
Other	14	(2.1)	0	(0.0)	0	(0.0)	0	(0.0)	0	(0.0)

Frequencies and percentages of males and females across recruitment sources for CHR participants are presented in Table 4. The most common recruitment source for both male and female participants was self-referral, accounting for 37.7% of females and 34.4% of males, followed by child and youth services, contributing 26.1% of females and 19.4% of males. A chi-square test of independence was conducted to examine the association between sex (male vs female) and recruitment source; the test revealed a statistically significant relationship (χ^2^ (12, *n* = 1640) = 42.88, *P* < .001). Standardized residuals (adjusted z-scores) indicated notable sex-specific patterns. Relative to the female-to-male ratio across the total sample, females were significantly underrepresented among referrals from psychiatric hospitals or departments (standardized residual = −2.8) and significantly overrepresented among those referred from child and youth services (standardized residual = 1.7). Conversely, males were overrepresented in psychiatric hospital or department referrals (standardized residual = 3.7) and underrepresented in referrals from child and youth services (standardized residual = −2.2).

**Table 4:** Percentages of Males and Females within Recruitment Sources for Clinical High-Risk Participants

**Recruitment source**	**Females (*n* = 1056)**		**Males (*n* = 584)**	
	**Frequency**	**(%)** ^**a**^	**Frequency**	**(%)** ^**a**^
Self-referred	398	(66.4)	201	(33.6)
Child and youth services	278	(71.1)	113	(28.9)
Psychiatric hospital or department	102	(49.0)	106	(51.0)
Another study	104	(60.5)	68	(39.5)
Adult community mental health services	45	(57.0)	34	(43.0)
General population screening	34	(68.0)	16	(32.0)
Community agencies	36	(73.5)	13	(36.5)
Physician	21	(65.6)	11	(34.4)
Non-physician	14	(56.0)	11	(44.0)
Post-secondary education	6	(54.5)	5	(45.5)
Grade school	3	(42.9)	4	(57.1)
Consumer organization	2	(66.7)	1	(33.3)
Other	13	(92.9)	1	(7.1)

a Percentages for males and females are percentages within recruitment source.

Means and standard deviations for age, total PSYCHS scores, and GF: Social scores for each recruitment source are presented in Table 5. In terms of age, the youngest participants were from child and youth services, community agencies, physician referrals, and grade school settings, all of whom had mean ages under 20 years. Regarding PSYCHS severity ratings, participants recruited through general population screening had the lowest severity ratings, while those recruited through adult community mental health services, consumer organizations, community agencies, and non-physician referrals had the highest PSYCHS ratings. Social functioning scores varied widely across recruitment sources. Participants from grade school, community agencies, and adult community mental health services reported the poorest ratings, while those from consumer organizations and physician referrals demonstrated relatively better social functioning ratings.

**Table 5:** Age, Attenuated Psychotic Symptoms, and Social Functioning Among Recruitment (Sources for Clinical High-Risk Participants)

**Recruitment source**	**CHR**	**Age (years)**	**PSYCHS total**	**GF:S**
	***n***	**Mean (SD)**	**Mean (SD)**	**Mean (SD)**
Self-referred	599	22.69 (3.59)	18.92 (8.86)	7.47 (1.20)
Child and youth services	391	18.79 (3.30)	18.77 (10.19)	6.98 (1.34)
Psychiatric hospital or department	208	21.67 (4.27)	17.92 (8.70)	6.70 (1.55)
Another study	172	21.83 (3.69)	17.65 (8.19)	7.51 (1.53)
Adult community mental health services	79	21.27 (3.69)	23.43 (11.14)	6.15 (1.50)
General population screening	50	23.20 (3.02)	16.60 (9.49)	7.50 (1.63)
Community agencies	49	17.76 (3.53)	23.00 (8.44)	6.13 (1.67)
Physician	32	18.61 (4.03)	20.69 (7.79)	7.83 (1.47)
Non-physician	25	20.04 (4.46)	23.00 (9.33)	7.00 (7.01)
Post-secondary education	11	22.39 (3.85)	19.73 (6.99)	7.75 (0.96)
Grade school	7	16.42 (1.86)	21.71 (15.56)	6.00 (1.00)
Consumer organization	3	24.97 (3.56)	27.33 (3.79)	9.00 (0.00)
Other	14	23.30 (4.30)	17.64 (8.79)	8.60 (0.55)

Abbreviations: GF:S, global functioning: Social Scale; PSYCHS, Positive Symptoms and Diagnostic Criteria for the CAARMS Harmonized with the SIPS.

A GLM was conducted to assess whether there were statistically significant differences in age, PSYCHS total scores, and GF: Social scores across the top 5 recruitment sources. The overall models were statistically significant for age (F(4,1444) = 69.94, *P* < 0 .0001), PSYCHS total scores, F(4,1443) = 6.07, *P* < .0001, and GF: Social scores F(4,1250) = 21.85, *P* < .0001. These results indicate that mean scores on all 3 measures differed significantly across at least 1 of the recruitment source groups.

Post hoc comparisons from the GLM analyzing the top 5 recruitment sources revealed several statistically significant differences in mean age, PSYCHS total scores, and GF: Social scores. Detailed pairwise comparisons are presented in Table 6. A supplementary analysis, which combined the remaining recruitment sources into a single category, confirmed that the patterns observed in the top 5 analyses were consistent across the broader dataset.

**Table 6:** Comparison of Top 5 Recruitment Sources of Clinical High-Risk Participants on Age, Attenuated Psychotic Symptom Severity, and Social Functioning

**Comparisons**	**Mean difference**	**95% CI**	***P*-value**
**Age**			
Self-referred vs another study	0.87	(0.01, 1.72)	^*^
Self-referred vs adult community mental health	1.42	(0.24, 2.61)	^*^
Self-referred vs psychiatric hospital/department	1.43	(0.63, 2.22)	^*^
Self-referred vs child/youth	3.90	(3.26, 4.55)	^*^
Child/youth another study	3.04	(2.13, 3.94)	^*^
Child/youth vs adult community mental health	2.48	(1.26, 3.70)	^*^
Child/youth vs psychiatric hospital/department	2.48	(1.63, 3.33)	^*^
**PSYCHS total**			
Adult community mental health vs self-referred	4.51	(1.47, 7.54)	^*^
Adult community mental health vs child/youth	4.66	(1.53, 7.79)	^*^
Adult community mental health vs psychiatric hospital/department	5.51	(2.16, 8.86)	^*^
Adult community mental health vs another study	5.79	(2.34, 9.23)	^*^
**Global functioning: social scale**			
Self-referred vs child/youth	0.44	(0.18, 0.69)	^*^
Self-referred vs psychiatric hospital/department	0.86	(0.55, 1.17)	^*^
Self-referred vs adult community mental health	1.03	(0.58, 1.48)	^*^
Another Study vs psychiatric hospital/department	0.70	(0.32, 1.08)	^*^
Another Study vs adult community mental health	0.87	(0.37, 1.37)	^*^
Child/youth vs psychiatric hospital/department	0.42	(0.09, 0.75)	^*^
Child/youth vs adult community mental health	0.59	(0.13, 1.05)	^*^

^*^ *P* < .05 (Tukey–Kramer corrected).

Abbreviations: Adult community mental health, adult community mental health services; Child/youth, child and youth services; Psychiatric hospital/department, psychiatric hospital or department of psychiatry within a hospital.

## Discussion

Our results show that participants in the AMP SCZ program were recruited from 13 different sources. Comparing current results to past studies is difficult due to limited referral data. However, we confirm that mental health settings and self-referral are common sources,^6^^,^^9^^,^^22^ particularly in the NAPLS consortium, where self-referral was most frequent, followed by mental health professionals and settings, physicians, and healthcare workers.^23^^,^^24^ The main difference is here there is a relative increase in the percentage of self-referrals.

As expected, differences were observed between the CHR and the CC groups. The majority of the CC participants were either self-referred or came from other studies. For CHR participants, the most common recruitment sources included self-referrals, child and youth services, psychiatric hospitals or psychiatric departments, and other studies. A key difference between the 2 networks was that the PRESCIENT network contributed a much larger proportion of the participants referred from child and youth services.

Regional differences in recruitment patterns were observed across continents. In North America, the majority of participants were self-referred, with additional recruitment distributed across a range of other sources. A significant proportion was also referred from other studies conducted at individual sites. In Australia, all PRESCIENT participants were recruited from Melbourne, with the primary recruitment source being child and youth services, followed by self-referrals. This reflects the development of youth mental health programs in Australia over the past 20 years, such as Headspace, which offers services for individuals aged 12-25. In Europe, most participants were referred from psychiatric hospitals and departments, followed by adult community mental health services. In Asia, participants were recruited through 3 primary sources: general population screening (a strategy unique to the region), psychiatric hospitals or departments, and self-referral, each contributing more or less equally to the Asian sample.

Self-referral, which included multiple recruitment strategies, was the most common recruitment source overall and was relatively high across most continents, except for Europe. The self-referred participants typically learned about the study through community-based online advertisements or, less commonly, through flyers, print advertisements, or public transit. These advertisements were often posted on mainstream social media platforms such as Facebook, Instagram, and Craigslist. Additionally, there are some newer digital platforms designed specifically for research recruitment, such as BuildClinical, that were successfully used in North America. For example, BuildClinical is a data-driven tool to help researchers with participant enrollment. BuildClinical generates self-referred participant leads that are potentially good matches for enrollment. Advertisements are run on various platforms in order to attract the target population. These advertisements are created in collaboration with study sites based on the specifics of the study and in adherence to the guidelines and procedures of Institutional Review Boards (IRB) and Research Ethics Boards. Potential participants view these advertisements and click to be redirected to a study-specific landing page where they complete an online pre-screen questionnaire. All self-referral leads are categorized as potentially eligible or ineligible depending on their responses, and sites are then able to follow up with eligible participants to consent and conduct a screening assessment.

There are several possibilities for the increase in self-referral. Possible changes in help-seeking practices, particularly with the increased use of the internet and social media (potentially accelerated by the COVID-19 pandemic), may have influenced recruitment patterns. It is also possible that the exclusion criterion of antipsychotic use may have reduced the percentage of help-seeking CHR in North America as compared to prior studies and consortia that did not have this exclusion criteria.

In examining demographic differences among CHR participants by recruitment source, it was found that although there were more females than males overall, the distribution of key recruitment sources was fairly similar between sexes. The exceptions were that females were overrepresented among referrals from child and youth services, which suggests that females may be more willing to seek help at such services. It has previously been noted that the composition of Melbourne youth mental health clinics has leaned toward females.^25^^,^^26^ A recent review suggested that help-seeking in psychosis is associated with being female.^27^ Males were overrepresented among referrals from psychiatric hospitals and psychiatric departments. This may indicate that males tend to delay help-seeking, potentially presenting with more severe or acute symptoms, or that they are less likely to use other resources to find help.

In examining differences in age, APS, and social functioning, analyses focused on the top 5 recruitment sources. As expected, individuals who self-referred—most often through internet searches—were significantly older than those in all other recruitment groups. In contrast, participants referred from child and youth services were noticeably younger than participants in 3 of the 4 other groups. The main finding, however, is that participants referred from adult community mental health services had significantly more severe APS, as rated by the PSYCHS, compared to all other recruitment sources. Most of these individuals came from subcategories such as early psychosis, early detection, or at-risk for psychosis clinics and programs. Notably, participants referred from adult community mental health services also had the lowest ratings on social functioning, performing more poorly than those who were self-referred, referred from child and youth services, or recruited through other studies. In contrast, self-referred participants and those who came from other studies had the highest ratings on social functioning. Overall, self-referred individuals were typically older, with less severe APS and higher levels of functioning, whereas those from adult community mental health services had poorer functioning and more severe APS. The remaining recruitment groups generally fell between these 2 extremes.

Previous research has suggested that transitions to psychosis are more likely among individuals recruited through clinical services.^4^ Although preliminary, it may be that those who were referred from clinical services and who are already demonstrating poorer functioning and more severe symptoms may have the poorest outcomes. Although, given the increasing number of self-referrals—many in response to online and social media outreach—it will be critical to examine, by the study’s conclusion, the outcome of those participants. The strength of this study lies in its large international sample, which captures a broad range of recruitment practices across diverse healthcare systems. However, there are several limitations. First, the data we used from this third release are cross-sectional, limiting conclusions about causality or changes over time. A second limitation involves potential misclassification of recruitment sources. While sites were provided with a predefined list (see Table 1), some participants may not have fit clearly into any category, leading raters to select the closest available option. As such, some recruitment pathways may have been underrepresented or mischaracterized. Training could have been provided to study sites, as was done for other measures, to ensure a clearer understanding of each recruitment source category. Such training might also have prompted the identification of additional, previously unrecognized recruitment sources.

Third, there are variations in clinical service infrastructure across countries (e.g., availability of specialized youth programs, community-based clinics, or differing structures of clinical services), such that recruitment sources vary by site. Despite these challenges, when combining all referrals from mental health services (child and youth services, adult community mental health services, psychiatric hospitals, and referrals from psychiatrists), these sources account for 766 participants, or 46.7% of the CHR sample. In comparison, 36.5% were self-referred, and 16.8% referred through non-mental health services or professionals. In summary, the findings presented here offer a comprehensive overview of recruitment sources in this large, international study. These insights are particularly valuable for the AMP SCZ program, as they can inform the planning of future pharmacological treatment trials by identifying the most effective recruitment sources and strategies. Moreover, with future data releases, it will be possible to examine participants from different recruitment sources in greater detail, including their associations with other clinical variables and risk factors. Further investigation into specific recruitment pathways, such as the type of social media platforms used for recruitment, will be informative. Importantly, the longitudinal nature of the project will allow for the evaluation of clinical trajectories across recruitment sources, helping to determine whether certain sources are more predictive of transitions to psychosis.

## Supplementary Material

Supplementary_Material_revised_sgaf013

## Data Availability

Data presented in this paper are available in the National Institute of Mental Health (NIMH) Data Archive (NDA). On the NDA site (NIMH Data Archive—AMPSCZ), there is information about available data and how it can be obtained.
