# Supplementary material for: Sample Ascertainment and Recruitment Sources in the Accelerating Medicines Partnership Schizophrenia Program
Source: Schizophr Bull Open. 2025 Aug 25;6(1):sgaf013. doi: 10.1093/schizbullopen/sgaf013 (PMC12377801; doi:10.1093/schizbullopen/sgaf013)

**Supplementary Table 1: Inclusion and Exclusion Criteria**

| **Inclusion Criteria** |
| --- |
| *CHR and Community Control (CC) inclusion criteria:* |
| (a) aged 12-30 years inclusive  (b) ability to give informed consent (parental/guardian consent obtained for participants aged <18 years) |
| *CHR only inclusion criteria:* |
| (c) CAARMS-defined (Trait Vulnerability; Attenuated Psychotic Symptoms; Brief Limited Intermittent Psychotic Symptoms) or Structured Interview for Psychosis-Risk Syndromes-defined (Brief Intermittent Psychotic Syndrome Current Progression; Attenuated Positive Symptom Syndrome Current Progression; Genetic Risk and Deterioration Current Progression) diagnostic criteria for CHR, determined using the Positive SYmptoms and Diagnostic Criteria for the CAARMS Harmonized with the SIPS (PSYCHS). |
| **Exclusion Criteria** |
| *CHR and Community Control exclusion criteria:* |
| (a) antipsychotic medication exposure equivalent to a total lifetime haloperidol dose of >50 mg, estimated based on available information, or current antipsychotic medication at time of baseline assessment,  (b) documented history of intellectual disability,  (c) past or current clinically relevant central nervous system disorder,  (d) traumatic brain injury rated 7 or above on the Traumatic Brain Injury screening instrument, or  (e) current or past psychotic disorder. |
| *Community Control only exclusion criteria:* |
| (f) meet CHR criteria or have a current or past Cluster A personality disorder,  (g) receiving any current treatment with psychotropic medication,  (h) family history (in first-degree relatives) of psychotic spectrum disorders. |

**Supplementary Table 2. Study sites for the AMP SCZ Program**

| **PRONET** | **PRESCIENT** |
| --- | --- |
| ***28 international sites*** | ***15 international sites, including the hub site in Melbourne, Orygen, which consists of the Orygen Specialist Program and 5 headspace clinics*** |
| **North America** | **Australia** |
| University of Calgary, Canada | Orygen, Melbourne – 6 sites |
| Douglas Research Centre, Montreal, Canada | **Europe** |
| University of California at Los Angeles | Department of Psychiatry and Psychotherapy, Jena University Hospital, Jena, Germany |
| University of California at San Diego | Copenhagen Research Centre for Mental Health, Copenhagen, Denmark |
| University of California at Irvine | Faculty of Medicine and University Hospital, Cologne, Germany |
| University of California at San Francisco | Treatment & Early Intervention in Psychosis Program, Lausanne University Hospital, Lausanne, Switzerland |
| University of Oregon, Oregon | Institute for Mental Health, University of Birmingham, Birmingham |
| Harvard/BIDMC, Massachusetts | **Asia** |
| Northwell Health, New York | Department of Psychiatry, Chonnam National University Medical School, Gwangju, South Korea |
| Mt. Sinai Hospital, New York | Institute of Mental Health, Singapore |
| University of Rochester, New York | LFK Faculty of Medicine, University of Hong Kong, Hong Kong |
| University of North Carolina at Chapel Hill | **South America** |
| Yale University, Connecticut | IMHAY, University of Chile, Santiago, Chile |
| Hartford Health Care, Connecticut |  |
| University of Pennsylvania |  |
| University of Pittsburgh, Pennsylvania |  |
| Temple University, Pennsylvania |  |
| Northwestern University, Illinois |  |
| University of Georgia, Atlanta |  |
| Washington University, Missouri |  |
| Ohio State University, Ohio |  |
| **Europe** |  |
| University of Pavia, Italy |  |
| Hospital General Universitario Gregorio, Maranon, Madrid, Spain |  |
| Ludwig-Maximilian-University, Munich, Germany |  |
| King's College, London |  |
| Cameo, Early Intervention Service, Cambridge |  |
| **Asia** |  |
| Shanghai Jiaotong University School of Medicine, China |  |
| Seoul National University, Korea |  |

| **Supplementary Table 3. Sociodemographic Variables at Baseline** | | | | | | | | |
| --- | --- | --- | --- | --- | --- | --- | --- | --- |
|  | | **CHR**  **N=1642** | | **Community Control**  **N=519** | | **Test statistic** | **Significance value** | **Effect**  **size** |
|  | | *Mean (SD)* | | *Mean (SD)* | | *t* | *p* | *Cohens d* |
| Age | | 21.2 (4.0) | | 21.6 (3.6) | | -2.24 | 0.025 | 0.105 |
| Years of education | | 13.0 (2.8) | | 13.9 (2.7) | | -6.84 | <.0001 | 0.327 |
|  | | *Frequency (%)* | | *Frequency (%)* | | *X^2^* | *p value* |  |
| Sex at birth | |  | |  | |  |  |  |
|  | Male | 585 | (35.63) | 200 | (38.54) | 1.44 | 0.2298 |  |
|  | Female | 1057 | (64.37) | 319 | (61.46) |  |  |  |
| Racial identification ^a^ | |  | |  | |  |  |  |
|  | Indigenous Groups | 19 | (1.20) | 4 | (0.78) | 56.40 | <.0001 |  |
|  | Native Hawaiian or Pacific Islander | 5 | (0.31) | 0 | (0.00) |  |  |  |
|  | East Asian | 139 | (8.75) | 91 | (17.74) |  |  |  |
|  | South Asian | 78 | (4.91) | 49 | (9.55) |  |  |  |
|  | Southeast Asian | 92 | (5.79) | 27 | (5.26) |  |  |  |
|  | Black | 137 | (8.63) | 32 | (6.24) |  |  |  |
|  | West/Central Asian and Middle Eastern | 52 | (3.27) | 8 | (1.56) |  |  |  |
|  | White | 925 | (58.25) | 268 | (52.24) |  |  |  |
|  | Multiracial | 141 | (8.88) | 34 | (6.63) |  |  |  |
| Marital status | |  | |  | |  |  |  |
|  | Single/never married | 1269 | (77.81) | 382 | (73.89) | 5.16 | 0.1598 |  |
|  | In a Relationship | 316 | (19.37) | 123 | (23.79) |  |  |  |
|  | Married/common law | 44 | (2.70) | 11 | (2.13) |  |  |  |
|  | Divorced | 2 | (0.12) | 1 | (0.19) |  |  |  |
| Living arrangements ^b^ | |  | |  | |  |  |  |
|  | With Family/Spouse | 951 | (58.38) | 254 | (49.22) | 17.95 | 0.0013 |  |
|  | Independent | 622 | (38.18) | 248 | (48.06) |  |  |  |
|  | Supported residential | 12 | (0.74) | 3 | (0.58) |  |  |  |
|  | Without housing | 8 | (0.49) | 0 | (0.00) |  |  |  |
|  | Other | 36 | (2.21) | 11 | (2.13) |  |  |  |
| **^a^East Asian includes: Chinese, Japanese, Korean; South Asian includes: Cambodian, Indonesian, Vietnamese; Southeast Asian includes: Indian, Pakistani, Sri Lankan; West/Central Asian and Middle Eastern includes: Egyptian, Lebanese, Emiratis, Afghans, Iranian.**  **^b^Independent includes living alone, with roommates; Other includes unknown, with another adult or student accommodations.** | | | | | | | | |

**Legend for Supplementary Figures**

***In all figures the x-axis is the top 5 referral sources***

1 Self-referral

2 Child and youth services

3 Psychiatric hospital or psychiatric department in a hospital

4 Another study

5 Adult community mental health services

***In the figures the y-axis is***

Fig 1 age in years

Fig 2 PSYCHS total scores – higher scores = more severe ratings

Fig 3 Global Functioning: Social – lower scores = poorer functioning

**Supplementary Figure 1. Distribution of Age in Years**


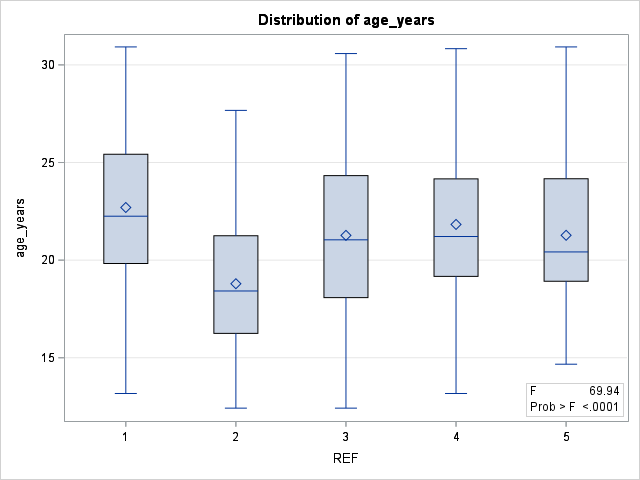


**Supplementary Figure 2. Distribution of Positive Psychotic Symptoms**


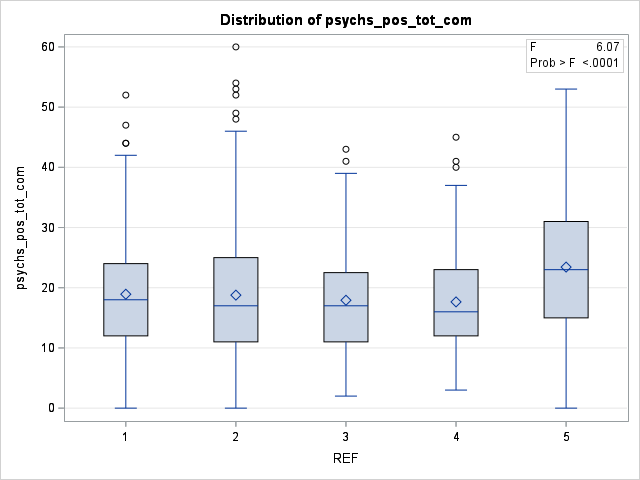


**Supplementary Figure 3. Distribution of Social Functioning**


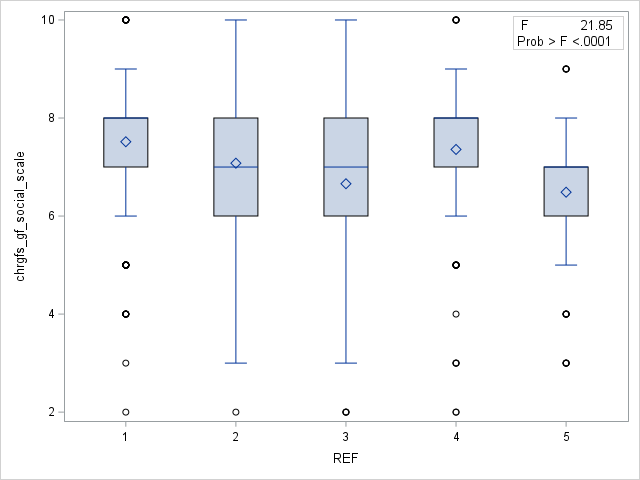

Supplement: Supplementary_Material_revised_sgaf013 [file supplementary_material_revised_sgaf013.docx]
